# Increased microvascular vasodilation and cardiovascular risk following a pre‐eclamptic pregnancy

**DOI:** 10.14814/phy2.12217

**Published:** 2014-11-26

**Authors:** Malia S. Q. Murphy, Meera Vignarajah, Graeme N. Smith

**Affiliations:** 1 Department of Biomedical and Molecular Science, Queen's University, Kingston, Ontario, Canada; 2 Department of Obstetrics and Gynecology, Queen's University, Kingston, Ontario, Canada

**Keywords:** Cardiovascular risk, microvascular function, pre‐eclampsia

## Abstract

Women who develop pre‐eclampsia are at high‐risk for premature cardiovascular disease and death. The aim of this study was to assess microvascular function and cardiovascular risk in the early postpartum period for women who did/did not have a pregnancy complicated by pre‐eclampsia. Peripheral microvascular function was assessed in women in the third trimester of uncomplicated pregnancies, with re‐evaluation at 2 and 6 months postpartum. The effect of pre‐eclampsia on postpartum microvascular function was assessed 2 and 6 months after delivery. Never‐pregnant, naturally cycling women served for comparison. Cutaneous microvascular reactivity to acetylcholine and sodium nitroprusside, delivered locally by iontophoresis, was measured by laser Doppler flowmetry. 30‐year and lifetime risk estimates for cardiovascular disease were established. Acetylcholine‐mediated vasodilation was enhanced by normotensive pregnancy, and declined to nonpregnant levels by 6 months postpartum. Acetylcholine‐mediated vasodilation remained high in pre‐eclamptic subjects from 2 to 6 months postpartum compared to normotensive and never‐pregnant controls. Pre‐eclamptic subjects exhibited elevated 30‐year and lifetime risk at 6 months postpartum. This study provides in vivo evidence of microvascular and cardiovascular risk implications of pre‐eclampsia as early as 6 months postpartum, and suggests that the development of pre‐eclampsia may be used to identify women at risk and eligible for risk screening and intervention.

## Abstract (precis)

This study demonstrates that microvascular function is altered by normal pregnancy, and returns to nonpregnant physiological levels by 6 months postpartum. In contrast, we report novel evidence of persistent augmentations in endothelial dependent vasodilation in a group of women monitored at 6 months postpartum of pre‐eclampsia. These findings provide support for the need of targeted postpartum period cardiovascular risk screening in this population of women.

## Introduction

Pre‐eclampsia (PE) is a complication of pregnancy characterized by widespread maternal endothelial dysfunction and *de‐novo* onset of hypertension and proteinuria after 20 weeks gestation. Endothelial dysfunction is common to the pathophysiology of both PE and cardiovascular disease (CVD), and it is now well‐established that the development of PE identifies women at increased risk for hypertension, ischemic heart disease, stroke, and premature death from CVD compared to women with an uncomplicated obstetrical history (Brown et al. 2013). While the symptoms of PE typically remit following delivery of the placenta, maternal vascular dysfunction has been shown to persist decades into the postpartum period (Ramsay et al. 2003). The risk associated with PE may be measured as early as a few months after the affected pregnancy (Smith et al. 2009, 2012), although the degree of recovery of maternal vascular function in the early postpartum period has not been well‐examined.

States of cardiovascular risk and endothelial dysfunction are classically associated with reduced or impaired endothelial‐dependent vasodilation in the larger resistance vessels; a matter that has been confirmed in PE subjects (Agatisa et al. 2004; Yinon et al. 2010). The means by which the vascular endothelium responds to pharmacologic stimuli has been demonstrated to vary by vascular bed, vessel size and by disease state, however. Indeed, examination of microvascular function during pregnancy suggests a negative correlation in vasodilator responses between the macro‐ and microcirculation, and endothelial‐dependent vasodilation is increased in the cutaneous microvessels of PE subjects (Davis et al. 2001; Blaauw et al. 2005; Khan et al. 2005). Despite a growing understanding of the importance of PE in identifying long‐term maternal cardiovascular health risks, over what time course these findings return to “normal” postpartum, if at all, is unknown. It has long been stated that the cardiovascular system, whether following a normal pregnancy or one complicated by PE, normalizes to the pre‐pregnant state by 6 weeks postpartum although little work has been done to study this. Multiple studies have now been done postpartum that report variable vascular dysregulation in formerly PE women (Chambers et al. 2001; Ramsay et al. 2003; Agatisa et al. 2004; Khan et al. 2005), however, all have been performed at a single time‐point ranging from months to years postpartum. Even within studies large ranges of time are used for analysis likely accounting for the significant variability in reported findings. There has been no attempt at focused assessment of changes in vascular function over the postpartum, either in formerly PE women, or controls. Given that the microcirculation is likely the initial site of development of vascular disease, we sought to determine whether previously documented increases in microvessel vasodilator responses in PE subjects would persist into the postpartum period.

## Materials and Methods

### Subject recruitment

This study was approved by the Queen's University Research Ethics Board. Written informed consent was obtained from all participants. Women experiencing normotensive singleton pregnancies were identified in the third trimester upon presentation for routine antenatal appointments at the Kingston General Hospital (KGH). PE subjects were identified through chart review, and defined as blood pressures ≥140/90 mmHg and proteinuria (≥300 mg/24 h or ≥30 mg/mmol albumin:creatinine random urine or ≥1+ on repeat dipstick). To determine if naturally cycling hormones or hormonal contraceptive use was relevant to postpartum measurements, microvascular function was assessed in never‐pregnant subjects. Never‐pregnant controls were studied three times over the course of a single menstrual cycle to correspond with menses (M), follicular (F) and luteal (L) phases. Estimation of phase was completed based on onset of menses and regular length of cycle. The medicated phase of monophasic oral contraceptive use was measured twice to correspond with time points of assessment in naturally cycling individuals. All individuals with a history of chronic hypertension, diabetes (including the development of gestational diabetes), renal disease, CVD, or current smoking were excluded.

Normotensive pregnant control subjects were examined in the third trimester and at 6 weeks and 6 months postpartum. Due to the variable effects of antihypertensive and antiseizure medications on vascular function it was not feasible to perform measurements in the time leading up to delivery for women with PE. For this reason PE subjects were only assessed at 6 weeks and 6 months postpartum. Participants still taking antihypertensive or antiseizure medications at follow‐up were excluded. At each visit blood pressure was measured and measures of obesity (waist circumference and body mass index [BMI]) were made.

At 6 months postpartum, normotensive and PE subjects were invited to attend the Maternal Health Clinic at KGH. Information captured included, weight, blood pressure, physical activity level, breastfeeding status, pregnancy history, family medical history of CVD, and current medication use. Blood requisitions were given for fasting blood and urine samples for core lab analysis of glucose, high‐sensitivity C‐reactive protein, lipid profiles, and albumin:creatinine ratio. All information from the maternal health clinic was used to generate CVD risk scores (Smith et al. 2013).

### Laser Doppler flowmetry

Measurements were taken in a temperature‐regulated environment with subjects lying in a semisupine position. Subjects were asked to abstain from caffeine consumption and use of over‐the‐counter medications the morning prior to testing. Cutaneous perfusion was measured by laser Doppler flowmetry (Moor Instruments Ltd., Axminster, UK). Two combined temperature and laser Doppler fluximetry probes surrounded by an iontophoresis Perspex chamber were secured to the volar aspect of the forearm. Iontophoresis chambers were adhered 4 cm apart, avoiding areas with broken skin and superficial veins. Continuous recordings of cutaneous perfusion and skin temperature were collected using a laser Doppler flow monitor (moorVMS‐LDF, Moor Instruments Ltd.) with data recorded in arbitrary perfusion units of flux (PU).

### Iontophoresis

**Figure 1.**
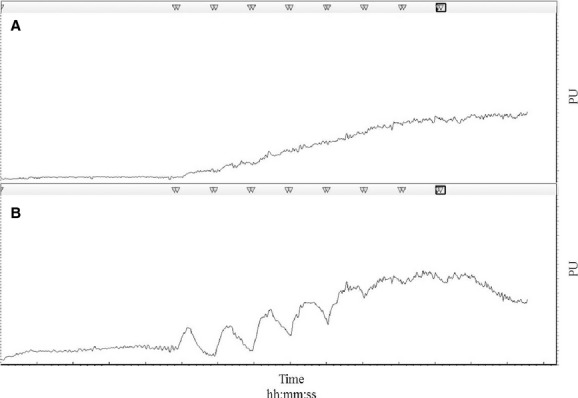
A representative depiction of dose‐response recordings generated from the application of laser Doppler Flowmetry and iontophoresis. Linear graphs correspond to changes in blood flow in response to (A) 1% SNP and (B) 1% ACh diluted in deionized water.

Iontophoresis is a technique used for the noninvasive delivery of drug solutions across the skin. The principle is based on the movement of charged ions across the skin in the presence of an applied electrical field. The magnitude of charge (Q) is dependent on size of the current (I), and corresponds to the amount of drug delivered. 1% solutions of acetylcholine (ACh; Miochol^®^‐E, Bausch&Lomb Inc., Vaughan, ON, Canada) and sodium nitroprusside (SNP; Nipride, Hospira Inc., Saint‐Laurent, QC, Canada) were introduced into the anodal and cathodal chambers for assessment of endothelial‐dependent and ‐independent function respectively. The vehicle for drug delivery was deionized sterile water. Following a 10‐min period of stable baseline perfusion recordings, dose‐response curves to ACh and SNP were obtained by the step‐wise application of currents (Davis et al. 2001) (5 *μ*A, 10 *μ*A, 15 *μ*A, 20 *μ*A, 50 *μ*A, and three applications of 100 *μ*A) by an iontophoresis controller (MIC2, Moor Instruments Ltd.). Currents were applied for 10 sec followed by 2 min recording periods for a total charge delivery of 4 mC. Corresponding changes in cutaneous blood flow was assessed using the laser Doppler flow monitor (moorVMS‐LDF, Moor Instruments Ltd.) (Fig. 1). Vasodilation was taken as the ratio of peak flux to an average of the total 10 min baseline perfusion for each drug administered per iontophoretic dose.

### Statistical analysis

Demographic variables are presented as mean ± standard deviation (SD). An unpaired *t*‐test or one‐way analysis of variance (ANOVA) with Bonferroni post hoc test was used to compare continuously distributed variables and a *χ*^2^ comparison was used for categorical measures. Dedicated software (moorVMS‐PC V3.1, Moor Instruments Ltd.) was used to analyze individual vascular responses to delivery of ACh and SNP by iontophoresis. Vasodilation was calculated as the ratio between the maximum achieved perfusion in response to each iontophoretic dose. Data were then exported to GraphPad Prism 5 (GraphPad Software, La Jolla, CA, www.graphpad.com) for statistical analyses and comparison within and between subject groups. Comparison within subject groups, across experimental time‐points were analyzed by matched two‐way ANOVA. Comparisons between subject groups, by time‐point of measurement were achieved by unpaired two‐way ANOVA. Statistical significance was accepted if the null hypothesis could be rejected at *P* < 0.05.

## Results

Baseline characteristics of all subjects are summarized in Table 1. Never‐pregnant subjects were younger than parous subjects, although did not differ by BMI when compared to prepregnant indices of both normotensive pregnant and PE subjects. Baseline perfusion and cutaneous temperature measurements did not differ significantly between subject group or across time (data not shown).

**Table 1.** Characteristics at time of recruitment or diagnosis of PE

	Never‐pregnant	Normotensive	Pre‐eclampsia
	*n* = 15	*n* = 23	*n* = 25
Age (year)	23.5 ± 3.5*	30.4 ± 4.2	32.1 ± 6.1
Parity, *n* (%)			
Primiparous	–	9 (39)	11 (48)
Multiparous	–	14 (61)	12 (52)
BMI (kg/m^2^)	22.2 ± 2.7	23.5 ± 3.5	26.1 ± 7.4
Pregnancy weight gain (kg)	–	14.8 ± 6.1	16.6 ± 8.6
Blood pressure (mmHg)			
Systolic	103.8 ± 6.2	112.5 ± 6.5	175.6 ± 21.6*
Diastolic	68.2 ± 7.6	72.3 ± 7.4	103.8 ± 8.2*
GA delivery (weeks)	–	39.8 ± 1.1	36.0 ± 3.8*

BMI, body mass index; SBP, systolic blood pressure; DBP, diastolic blood pressure.

* *P* < 0.0001 versus Normotensive group.

### Microvascular function

#### Effect of naturally cycling hormones and oral contraceptives on microvascular function

Twenty‐five (*n* = 25) never‐pregnant subjects participated in the study. Of these 15 (*n* = 15) were naturally cycling and 10 (*n* = 10) individuals reported use of monophasic oral contraceptive medication for a minimum of 4 months.

Measurements taken across the course of a natural menstrual cycle demonstrated no effect by phase on microvascular vasodilation. Similarly, oral contraceptive use did not alter microvascular measurements compared to naturally cycling individuals (data not shown). For this reason, data from normotensive and PE subjects was compared to the midfollicular phase of never‐pregnant subjects in subsequent figures.

#### Effect of normotensive pregnancy on microvascular function

**Figure 2.**
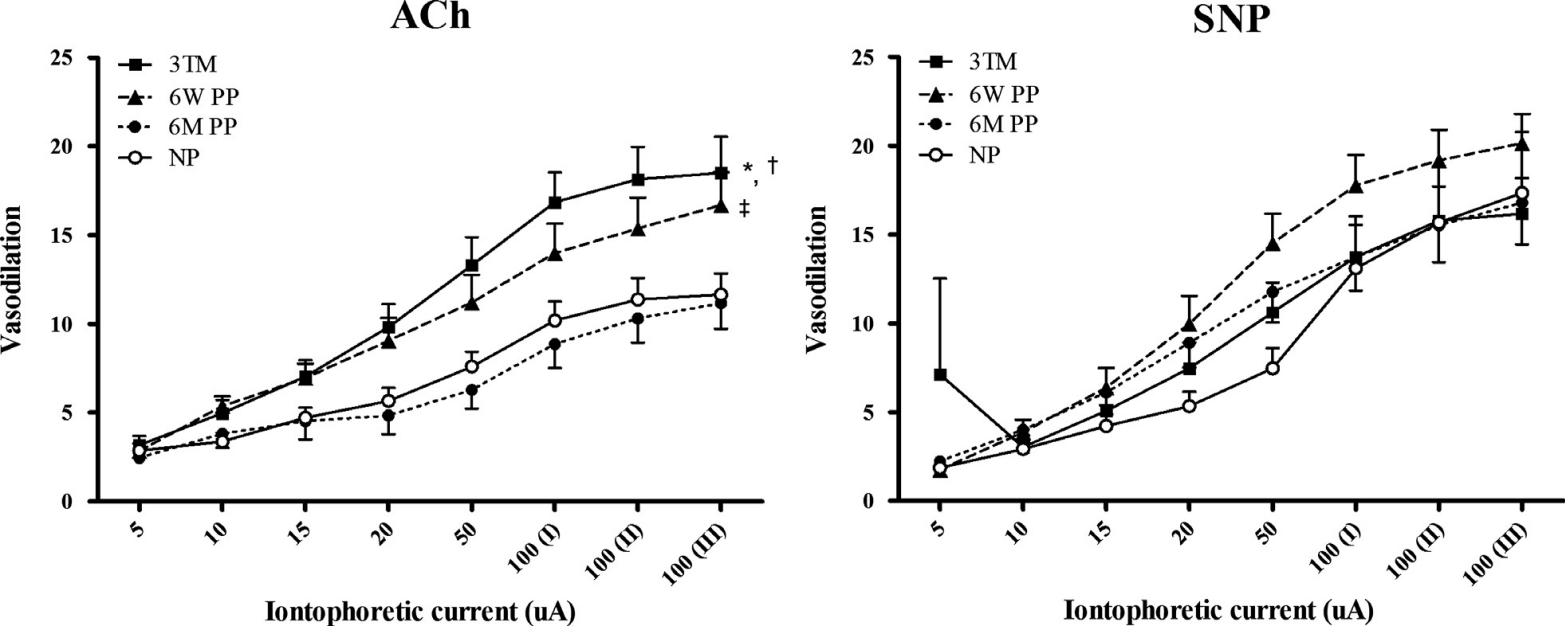
Endothelial‐dependent responses to ACh and endothelial‐independent responses to SNP in normotensive women (*n* = 23) in the third trimester (3TM), 6 weeks postpartum (6W PP) and 6 months postpartum (6M PP) compared to never‐pregnant (NP, *n* = 15) subjects. Data are presented as mean ± SEM. Post hoc comparisons*P < 0.001 3TM versus 6M PP; †*P* < 0.05 3TM versus NP; ‡*P* < 0.05, 6W PP versus 6M PP.

Microvascular measurements taken in 23 (*n* = 23) normotensive pregnant controls demonstrated that the third trimester of pregnancy was associated with enhanced endothelial‐dependent vasodilation in response to ACh (*P* < 0.0001) compared to never‐pregnant levels. This effect persisted at 6 weeks postpartum. Microvascular reactivity returned to levels similar to never‐pregnant controls by 6 months postpartum (*P* > 0.05). Microvascular responses to SNP were unchanged in pregnancy and in the postpartum (*P* > 0.05) (Fig. 2).

#### Effect of PE on postpartum microvascular function

**Figure 3.**
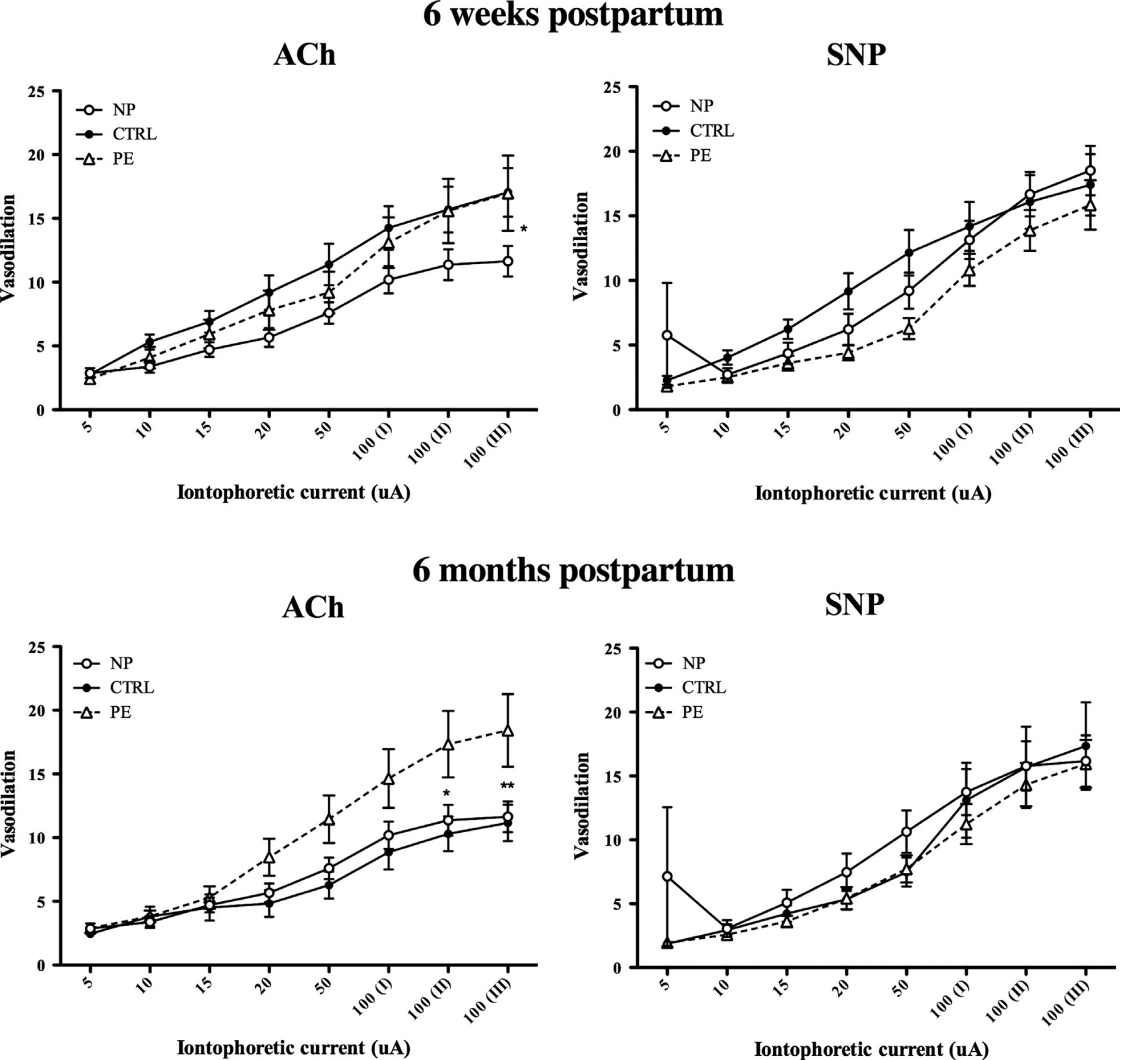
Comparison of microvascular responses at 6 weeks postpartum and 6 months postpartum to ACh and SNP. Data are presented as mean ± SEM. NP, never‐pregnant (*n* = 15); CTRL, normotensive control, (*n* = 23); PE at 6 weeks postpartum (*n* = 15), PE at 6 months postpartum (*n* = 25); posthoc comparisons*P < 0.05; **P < 0.01.

Microvascular reactivity to ACh and SNP was unchanged from 6 weeks to 6 months postpartum in 12 (*n* = 12) PE subjects. Maximal perfusion responses to ACh were not different at 6 weeks postpartum, but were significantly elevated in PE subjects compared to controls at 6 months postpartum. Postpartum vascular responses to SNP did not differ. To better assess the effect of PE on microvascular function at 6 months postpartum, data from an additional 10 PE subjects was included (Fig. 3). Baseline characteristics and microvascular measurements for these additional subjects did not differ from the initial PE group. For this reason, the additional data is included in Table 1 and Figure 3.

#### Cardiovascular risk at 6 months postpartum

Normotensive (*n* = 21) and PE (*n* = 22) subjects provided complete biophysical profiles at 6 months postpartum (CTRL 26.1 ± 3.8 weeks vs. PE 28.1 ± 2.6 weeks; *P* > 0.05). Postpartum measurements of obesity at 6 month postpartum did not differ between subject groups, although fewer PE subjects were breastfeeding at 6 months postpartum. 30‐year (Pencina et al. 2009) and lifetime (Lloyd‐Jones 2006) cardiovascular risk scores were based on physical and biochemical parameters of cardiovascular risk (sex, age, smoking, total cholesterol, fasting glucose, systolic blood pressure, antihypertensive use; and sex, smoking, total cholesterol fasting glucose, systolic blood pressure, diastolic blood pressure, antihypertensive usage, respectively) and calculated following the 6 months postpartum clinic assessment. A summary of findings are found in Table 2.

**Table 2.** Cardiovascular risk variables at 6 months postpartum

	Normotensive *n* = 21	Pre‐eclampsia *n* = 22	*P*‐value
Weeks postpartum (wks)	26.1 ± 3.8	28.1 ± 2.6	0.0844
BMI (kg/m^2^)	25.4 ± 4.7	29.4 ± 8.4	0.0674
Weight retention (kg)	5.6 ± 5.9	6.1 ± 9.5	0.8377
Waist circumference (cm)	85.7 ± 11.7	91.78 ± 18.74	0.2166
Breastfeeding, *n* (%)	17 (80.9)	12 (54.5)	0.0001
Blood pressure (mmHg)			
Systolic	106.2 ± 8.99	124.0 ± 13.2	*P* < 0.0001
Diastolic	70.14 ± 7.44	85.27 ± 9.76	*P* < 0.0001
Fasting glucose (mmol/L)	4.50 ± 0.38	4.68 ± 0.37	0.1345
Total cholesterol (mmol/L)	4.78 ± 0.98	4.72 ± 0.83	0.8010
Triglycerides (mmol/L)	0.66 ± 0.31	1.332 ± 1.2	0.0170
HDL cholesterol (mmol/L)	1.65 ± 0.4	1.37 ± 0.38	0.0232
LDL cholesterol (mmol/L)	2.83 ± 0.88	2.77 ± 0.71	0.8238
Albumin:Creatinine (mg/mmol)	0.49 ± 0.59	3.8 ± 7.8	0.0589
C‐reactive protein	2.69 ± 3.1	5.8 ± 10.46	0.993
Metabolic syndrome, *n* (%)	0 (0)	6 (27)	*P* < 0.00001
30 year risk	5.0 ± 1.85	10.0 ± 4.42	0.0002
Lifetime risk, *n* (%)			
Low risk (<39%)	13 (62)	9 (40)	0.0029
High risk (>39%)	8 (38)	13 (60)	

BMI, body mass index; SBP, systolic blood pressure; DBP, diastolic blood pressure; HDL, high density lipoprotein; LDL, low density lipoprotein.

Our PE group included five women with early‐onset PE, and 18 with late‐onset PE. Additionally 15 women experienced severe PE, while five experienced mild PE. Stratification of subjects revealed no differences in ACh‐mediated vasodilation by onset and severity of PE (data not shown). Conversely, high‐risk PE subjects had elevated vasoreactivity to ACh compared to high‐risk normotensive controls at 6 months postpartum (PE *n* = 10, vasodilation: 15.17 ± 2.207 vs. CTRL *n* = 7, vasodilation: 8.282 ± 1.671; *P* < 0.05). This difference was not observed when vasodilation was compared between low‐risk groups (PE *n* = 7, vasodilation: 17.41 ± 3.540 vs. CTRL *n* = 12, vasodilation: 12.12 ± 2.210; *P* > 0.05).

## Discussion

Here we describe for the first time, prospective assessment of postpartum recovery of microvascular function following uncomplicated and PE pregnancies. Our findings demonstrate that pregnancy‐enhanced endothelial‐dependent vasodilation returns to nonpregnant levels by 6 months postpartum. In contrast, ACh stimulated vasodilation remains persistently increased in the cutaneous microvessels of women with a recent history of PE such that by 6 months postpartum endothelial‐dependent vasodilation exceeds levels seen in women with uncomplicated obstetrical history and never‐pregnant controls.

Our findings in postpartum PE women are not consistent with those reported in larger vessels. Attenuated vascular responses to flow‐mediated dilation and venous occlusion plethysmography are reported months to years after afflicted pregnancies (Chambers et al. 2001; Agatisa et al. 2004; Lampinen et al. 2006; Yinon et al. 2010). A single evaluation of microvascular function 15–25 years after PE described reduced endothelial‐dependent and ‐independent responses typical of the larger conduit vessels (Ramsay et al. 2003).

Comparison of endothelial function in conduit arteries with that in small resistance vessels should be made with caution. Macro‐ and microvascular properties of vasodilation often poorly correlate, and even still the use of pharmacological (e.g. ACh, SNP) and physiological (shear stress) stimuli likely induce different contributions from the endothelium to prompt vasodilation (Dhindsa et al. 2008). Evaluation of microvascular function in response to pharmacologic stimuli in PE and the early postpartum are few, but indicate that microvascular vasodilator responses to ACh are increased in women with PE compared to those with uncomplicated pregnancy (Davis et al. 2001; Blaauw et al. 2005; Khan et al. 2005). Khan et al. (2005) performed serial measurements from 22 weeks gestation to 6 weeks beyond delivery in 54 control women and 15 women who developed PE. The authors reported increased vasodilator responses to ACh in PE women before delivery, which subsequently declined to levels comparable to controls by 6 weeks postpartum. In contrast, Blaauw and colleagues reported enhanced microvascular responses to ACh in subjects examined between 3 and 11 months postpartum (Blaauw et al. 2005). Our findings both corroborate and extend these observations by taking consecutive measurements of microvascular function into the postpartum period at well‐defined time‐points. In consideration of previous reports, we suggest that pregnancy‐mediated increases in endothelium‐dependent vasodilation declines postpartum following uncomplicated pregnancies, whereas this postpartum “recovery” of vascular function is altered in PE subjects. At what time microvascular responses transition from enhanced to attenuated, as reported by 15–25 year follow‐up of this population (Ramsay et al. 2003) is unclear however, and the mechanisms underlying these changes require further investigation. Even further, paired measurements of large and microvessel function in this population would be beneficial to enhance of our understanding of microvessel physiology in health and disease.

Differences in microvascular function are unlikely the result of increased sensitivity to nitric oxide by the vascular smooth muscle at the time points studied here, as responses to SNP were not altered in PE subjects. Rather, this points to changes in vasodilator synthesis or dependence, or perhaps in the permeability of the endothelium to vasoactive substances. The relative contribution of each of nitric oxide, prostaglandins, and endothelium derived hyperpolarizing factors to endothelial‐dependent vascular responses in the forearm microcirculation has been debated, although in vivo experimentation with eNOS and COX inhibitors suggests that cutaneous microvascular responses to ACh are likely the result of contributions from both nitric oxide and prostaglandin pathways (Khan et al. 1997; Dalle‐Ave et al. 2004; Holowatz et al. 2005; Kellogg et al. 2005; Medow et al. 2008). Demonstrated plasticity of endothelial function within the coronary circulation indicates a shift dependence of vasodilatory mediators in adult health versus disease (Beyer and Gutterman 2012). Therefore, there is the potential for similar response mechanisms modulating endothelial‐dependent activity at the level of the cutaneous microvessels in states of cardiovascular risk that may explain inconsistencies in correlation between brachial and cutaneous function (Hansell et al. 2004; Dhindsa et al. 2008).

Laser Doppler flowmetry as used in the present study provides a useful, noninvasive tool for assessment of microvascular function as it enables the evaluation of microvascular blood flow continuously over time, and in response to a given stimulus (Kubli et al. 2000; Hodges and Del Pozzi 2014). The inherent variability in Doppler and iontophoresis measurements, however, must be considered. Reported interindividual and day‐to‐day variation of flowmetry data may vary significantly and must be reported for each study (Cracowski et al. 2006; Roustit et al. 2010). The major source of variation is the site of measurement, however, and when spatial variability is minimized day‐to‐day reproducibility of Doppler flowmetry data compares well with traditional measurements of vascular function (<10%) (Kubli et al. 2000). This technique in our laboratory has shown good reproducibility (CV%30.5; ICC0.64), although the prolonged nature of follow‐up in our experiments made spatial standardization difficult. Given that variability in baseline flow in our subjects was minimal, the significant alterations in vasodilator responses detected in our cohort despite the high degree of variability in single‐point Doppler measurements in forearm microvessels are substantial.

Abnormalities in endothelial function contribute substantially to cardiovascular risk and outcome, and whether endothelial dysfunction is present before, or incurred as a result of pregnancies affected by PE remains an important question that needs to be addressed. It has been suggested that the development of PE may be in part due to the subclinical physiological state of a mother's endothelium prior to pregnancy, and its ability to meet the hemodynamic and metabolic demands of the growing conceptus. Indeed, subclinical endothelial dysfunction prior to a diagnosis of PE has been well‐described (Savvidou et al. 2003; Levine et al. 2004; Khan et al. 2005). Although our sample sizes were small, stratification of our data by cardiovascular risk lends support to this hypothesis. Differences in endothelial function between PE and control subjects were persistent only in women with high lifetime risk for CVD, suggesting that biophysical profiles play an important role in of the persistence or progression of altered resistance vessel function in women with a history of PE.

Risk of CVD is modifiable with lifestyle intervention, and modest modifications to dietary and exercise habit have demonstrable effects on blood pressure, lipid profiles and inflammatory and hemostatic regulators (Mora et al. 2007). A recent study from the Netherlands examined the benefits of 12 week exercise training in 6–12 month postpartum PE subjects. In addition to benefits on blood pressure, parameters of obesity and cholesterol, exercise training reduced carotid artery intima media thickness and improved brachial and femoral artery flow mediated dilation responses. Autonomic cardiac activity quantified by spectral analysis of heart rate variability was also improved following training. Interestingly, while training improved all parameters of endothelial function and autonomic activity, but did not normalize these parameters in formerly PE women to levels comparable to trained controls (Scholten et al. 2014). Pharmacologic intervention in nonhypertensive postpartum PE women with demonstrated risk for CVD may also prove a promising option for CVD risk intervention. One and five year follow‐up of newly diagnosed hypertensive patients prescribed daily use of ACE inhibitors demonstrates long‐term improvements in endothelial progenitor cell function, inversely correlated with carotid intimal media thickness (Cacciatore et al. 2011). Additionally, prescription of ACE inhibitors reduces albuminuria and progressive kidney failure in type 1 diabetes and improves endothelial‐dependent function (O'Driscoll et al. 1997; Arcaro et al. 1999).

In conclusion, this study compares longitudinal in vivo microvascular reactivity following normotensive and PE pregnancies. ACh‐mediated vasodilation was elevated in the postpartum PE period and PE was associated with increased endothelial‐dependent vasodilation compared to normotensive pregnancies at 6 months postpartum. While the resulting sample sizes were small, we feel that the findings presented here provide important evidence of the function changes taking place in the maternal endothelium in the early postpartum period following PE. These findings provide support for the need for targeted early postpartum cardiovascular risk screening in women who have experienced pregnancies complicated by PE.

## Acknowledgments

The authors would like to thank Michelle Roddy, RN, BScN, and Jessica Pudwell, MPH of Kingston General Hospital, for their assistance in subject identification and compilation of patient data. These findings have been presented at the 61st Annual Meeting, Society for Gynecologic Investigation in Florence, Italy (March 26–29 2014). The abstract was published in Reproductive Sciences. Murphy MSQ, G Smith. (2014) Pre‐eclampsia is associated with early postpartum endothelial dysfunction as measured by laser Doppler flowmetry and iontophoresis (O‐138). *Reproductive Sciences*. 2014; 21(3) Supplement.

## Conflict of Interest

The authors declare no conflict of interest.
